# Converging Patterns of Heterotrophic Respiration Between Growing and Non-Growing Seasons in Northern Temperate Grasslands

**DOI:** 10.3390/plants14162590

**Published:** 2025-08-20

**Authors:** Caiqin Liu, Honglei Jiang, Xiali Guo

**Affiliations:** 1 Institute of Science and Technology for Carbon Peak & Neutrality, Guangxi University, Nanning 530004, China; liucq1120@163.com; 2 College of Life Sciences, Sichuan University, Chengdu 610065, China; 3 Guangxi Laboratory on the Study of Coral Reefs in the South China Sea, Coral Reef Research Center of China, School of Marine Sciences, Guangxi University, Nanning 530004, China; 4 Guangxi Youyiguan Forest Ecosystem Observation and Research Station, Pingxiang 532600, China

**Keywords:** soil heterotrophic respiration, temperate grasslands, growing and non-growing seasons, eddy covariance, microbial respiration

## Abstract

Temperate grasslands are highly sensitive to climate change and play a crucial role in terrestrial carbon cycling. In the context of global warming, heterotrophic respiration (Rh) has intensified, contributing significantly to atmospheric CO_2_ emissions. However, seasonal patterns of Rh, particularly differences between the growing season (GS) and non-growing season (non-GS), remain poorly quantified. This study used daily eddy covariance data from multiple flux towers combined with MODIS GPP and NPP products to estimate Rh across temperate grasslands from 2002 to 2021. We examined interannual variations in GS and non-GS Rh contributions and assessed their relationships with key hydrothermal variables. The results showed that mean Rh during GS and non-GS was 527 ± 357 and 341 ± 180 g C m^−2^ yr^−1^, respectively, accounting for 57.8 ± 14.6% and 42.2 ± 14.6% of the annual Rh. Moreover, GS Rh exhibited a declining trend, while non-GS Rh increased over time, indicating a gradual convergence in their seasonal contributions. This pattern was primarily driven by increasing drought stress in GS and warmer, moderately moist conditions in non-GS that favored microbial activity. Our findings underscore the necessity of distinguishing seasonal Rh dynamics when investigating global carbon cycle dynamics. Future earth system models should place greater emphasis on seasonal differences in soil respiration processes by explicitly incorporating the influence of soil moisture on the decomposition rate of soil organic carbon, in order to improve the accuracy of carbon release risk assessments under global change scenarios.

## 1. Introduction

Grassland ecosystems are among the most widespread terrestrial vegetation types globally, covering approximately 46 million km^2^ and accounting for about 25–30% of the Earth’s land surface [1]. Grasslands provide essential ecological and productive functions, including soil and water conservation, wind erosion control, climate regulation, and water retention [2]. Temperate grasslands are home to a high biodiversity of mammals and birds, and store large quantities of carbon in their soil. Total carbon stocks are estimated at 450–550 Gt C, which constitutes approximately 18–31% of global terrestrial carbon, with a correspondingly high potential for carbon sequestration [1]. However, as both climate-sensitive and ecologically vulnerable systems, they are undergoing a gradual warming and drying trend under global change, leading to significant degradation and conversion [3,4]. This not only reduces biodiversity and ecosystem services, but also compromises their role as carbon sinks, thereby influencing global greenhouse gas dynamics [2,4].

Soil respiration (Rs), the process through which CO_2_ is emitted from soil to the atmosphere, represents the largest carbon flux between terrestrial ecosystems and the atmosphere, playing a pivotal role in the global carbon cycle [5,6]. Rs is typically divided into two components: heterotrophic respiration (Rh; from microbial decomposition of organic matter) and autotrophic respiration (Ra; CO_2_ release from live root metabolism). The global terrestrial Rs flux is estimated at 68–110 Pg C yr^−1^ [5,7,8,9], approximately ten times the current annual fossil fuel emissions (~11 Pg C yr^−1^) [5]. Rh is one of the primary mechanisms through which terrestrial ecosystems release CO_2_ into the atmosphere, and has shown an increasing relative contribution over the past two decades [10], estimated at 42–57 Pg C yr^−1^ [8,10,11]. Due to hydrothermal disturbances, the global mean surface Rh:Rs ratio increased from 0.54 in 1990 to 0.63 by 2014 [10].

Despite the well-documented seasonal dynamics of Rs (including Ra and Rh), existing studies have primarily focused on the seasonal response of annual total Rs to climate change [12,13,14]. These studies have shown that non-growing season (non-GS) Rs can contribute 2–37% of the annual total Rs [15], and that Rh during the non-GS contributes approximately 40% more to Rs than during the growing season (GS) [16]. This indicates that non-GS Rh may account for a larger share of the annual Rh and may result in higher CO_2_ emissions, making it a critical component in soil carbon flux assessments. However, current research on Rh has largely emphasized interannual variations in total Rh fluxes [10,11,12,17] and its ratio to various carbon fluxes (e.g., Rh:Rs, Rh:Ra, and Rh:GPP) [10,18,19], while studies that explicitly separate Rh into growing and non-growing season components and compare their behaviors are extremely limited. For instance, Gonzalez et al. [20], Wang et al. [21], and Bhupinderpal-Singh et al. [22] only analyzed GS Rh over one or two years, while investigations of non-GS Rh have mainly focused on forest ecosystems [15,16]. This limited availability of seasonal Rh studies may stem from the inherent challenges in long-term Rh monitoring. Although Rs can be readily measured using automated or manual chamber systems, partitioning Rs into Ra and Rh components remains methodologically complex and uncertain [23]. Conventional approaches often rely on isotope tracing or destructive techniques such as trenching or tree girdling, which are not well-suited for large-scale or continuous monitoring [24]. Although soil carbon models are capable of providing long-term Rh simulations, most existing studies primarily focus on cumulative annual Rh emissions, rather than seasonal dynamics [11,25,26]. However, understanding the respective contributions and temporal shifts of GS and non-GS Rh to annual Rh—and their responses to climatic drivers—is critical for constraining terrestrial carbon budgets. Yet, this remains poorly understood in grassland systems.

Using long-term eddy covariance flux data, combined with remotely sensed products and in situ hydrothermal variables from 19 temperate grassland sites, we analyzed the differences in Rh between the GS and non-GS. We first examined the interannual variation patterns of Rh contributions during the two seasons, followed by an assessment of their responses to different hydrothermal factors. Finally, we discussed the potential drivers underlying the observed changes in seasonal Rh contributions. Our results provide evidence that under the warming and drying trend, the seasonal patterns of heterotrophic respiration in temperate grasslands tend to converge between the growing and non-growing seasons.

## 2. Result

### 2.1. Seasonal Pattern of Hydrothermal Variables

**Figure 1:**
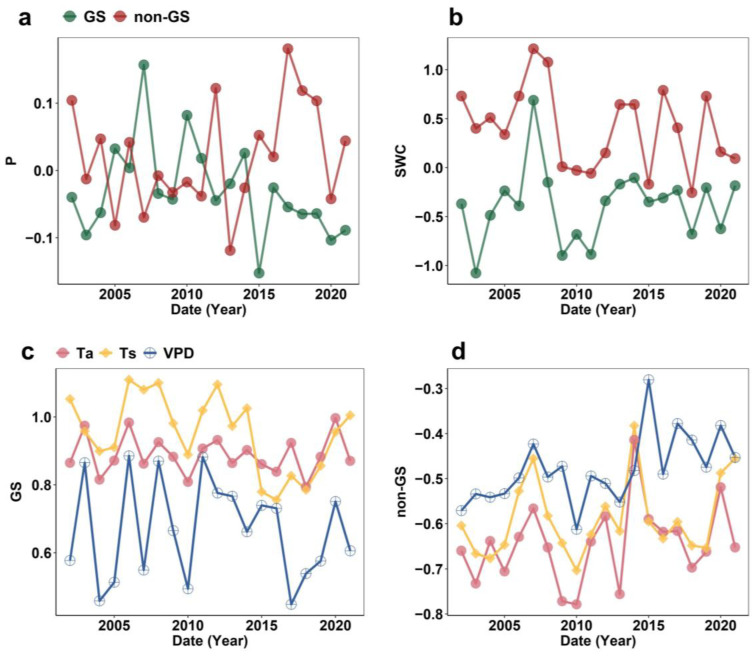
Interannual patterns of hydrothermal variables during the growing season (GS) and non-growing season (non-GS). (**a**,**b**) GS and non-GS of precipitation, respectively, (P) and shallow soil water content (SWC). (**c**,**d**) Interannual variations of air temperature (Ta), shallow soil temperature (Ts), and vapor pressure deficit (VPD) during GS and non-GS, respectively.

From 2002 to 2021, except for precipitation (P), the other four climate-related variables (air temperature, Ta; soil temperature, Ts; soil water content, SWC; and vapor pressure deficit, VPD) exhibited significant seasonal differences between the growing season (GS) and non-growing season (non-GS) (*p* < 0.001, Wilcoxon rank-sum test, Figure 1). Ta, Ts, and VPD were consistently higher in GS than in non-GS (Figure 1c,d), displaying low interannual variability, with slight upward trends in Ta and VPD during non-GS. SWC was higher in non-GS, fluctuating strongly in both seasons, and showing a clear downward trend over time (Figure 1a). Precipitation declined during non-GS until 2014 then increased, while GS precipitation followed an opposite trend (Figure 1b).

### 2.2. Seasonal Rh and Its Contribution to Annual Rh

**Figure 2:**
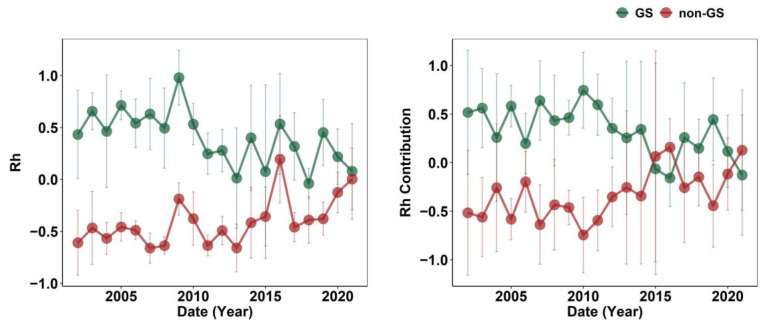
Interannual patterns of growing season (GS) and non-growing season (non-GS) Rh (**left**) and their contributions to annual Rh (**right**). The error bar is standard error.

A highly significant difference was observed between GS Rh and non-GS Rh, as determined by the Wilcoxon test (*p* < 0.001). GS Rh was overall higher than non-GS Rh, but their interannual trends moved in opposite directions (Figure 2). Specifically, non-GS Rh exhibited a steady increase over time—its contribution proportion gradually approached and even surpassed that of GS Rh, which displayed a declining trend. Over the past decade, the proportional contribution of non-GS Rh to annual Rh increased significantly, suggesting that non-GS heterotrophic respiration has become increasingly important in carbon emissions, reflecting heightened sensitivity of the non-GS carbon cycle to climate variability.

### 2.3. Seasonal Rh Contribution to Annual Rh in Spatial Difference

**Figure 3:**
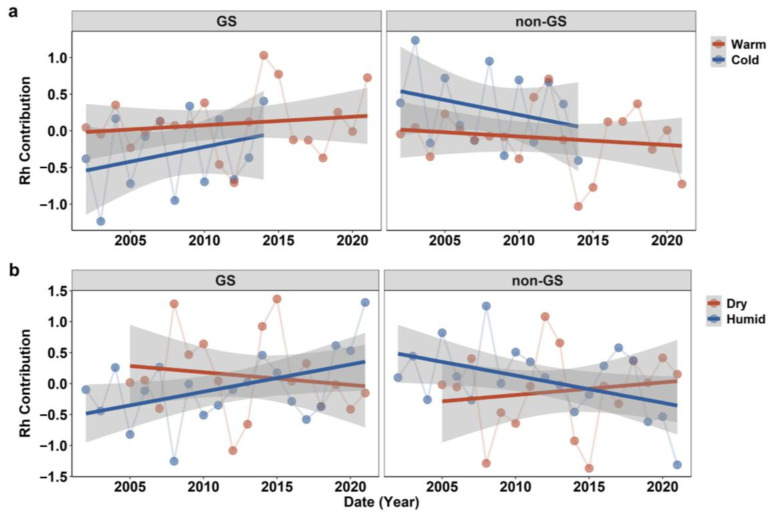
Temporal trends in Rh contribution in warm vs. cold regions (**a**) and dry vs. humid regions (**b**) during the growing season (GS, **left**) and non-growing season (non-GS, **right**). Regions are classified using site-level mean annual temperature (15 °C) and precipitation (220 mm) thresholds. Solid lines represent trend fits and shaded areas indicate the 95% confidence intervals.

Based on long-term mean annual temperature (15 °C) and precipitation (220 mm), the 19 sites were classified into relatively warm/cold and dry/humid regions. The GS Rh contribution showed a slight increase in warm and humid areas but a decline in dry regions, whereas non-GS Rh increased in dry regions and decreased in cold and humid areas (Figure 3). These contrasting patterns indicate that seasonal Rh partitioning is strongly modulated by regional hydrothermal conditions. GS Rh decline and non-GS Rh increase patterns occurred in arid regions.

### 2.4. Seasonal Rh Response to Hydrothermal Variables

**Figure 4:**
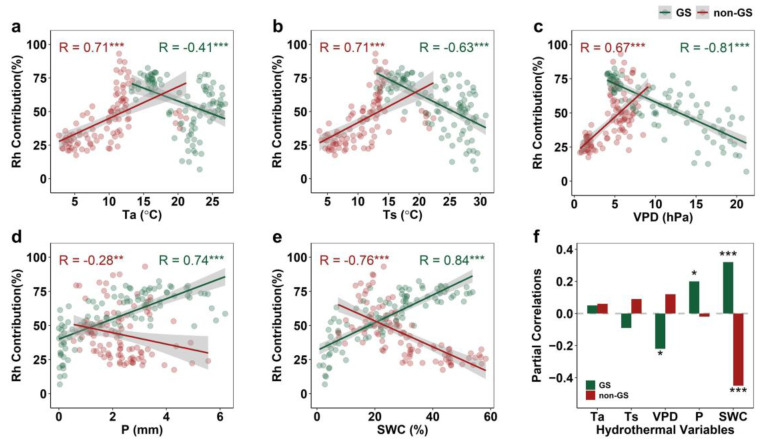
Linear relationships between Rh contribution and hydrothermal variables in the growing season (GS) and non-growing season (non-GS): (**a**) Ta, air temperature; (**b**) Ts, shallow soil temperature; (**c**) VPD, vapor pressure deficit; (**d**) P, precipitation; (**e**) SWC, shallow soil water content. (**f**) Partial correlations between Rh contribution and hydrothermal variables. *** denotes *p* < 0.001, ** denotes *p* < 0.01, * denotes *p* < 0.05.

Both GS and non-GS Rh contributions were significantly correlated with all hydrothermal variables, but with opposite seasonal patterns (Figure 4). In GS, Rh contribution was negatively related to Ta, Ts, and VPD (strongest for VPD, R = −0.81) and positively to P and SWC (R = 0.74, 0.84). In non-GS, Rh was positively related to Ta, Ts, and VPD (R = 0.71, 0.71, 0.67) but negatively to P and SWC (R = −0.28, −0.76). Partial correlation analysis (Figure 4f) showed that GS Rh contribution was mainly driven by positive effects of SWC and P and negative effects of VPD, while non-GS Rh contribution was significantly constrained by SWC (*p* < 0.001).

### 2.5. Importance of Hydrothermal Drivers for Seasonal Rh

**Figure 5:**
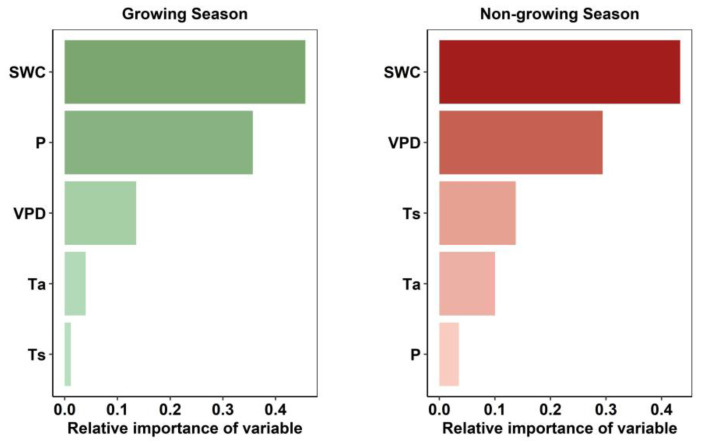
Relative importance of hydrothermal variables for Rh contribution during the growing season (GS) and non-growing season (non-GS).

The relative importance of hydrothermal drivers differed markedly between seasons. The coefficient of determination (R^2^) values were 0.81 and 0.67 for GS and non-GS, respectively, with corresponding root mean square errors (RMSEs) of 9.53% and 9.90%, respectively. During the GS, soil water content (SWC, 0.43) and precipitation (P, 0.36) were the dominant factors (Figure 5). In contrast, SWC (0.43) and vapor pressure deficit (VPD, 0.30) played leading roles during the non-GS (Figure 5). These findings are consistent with the partial correlation analysis results (Figure 4f), reinforcing the seasonal shifts in environmental controls on Rh.

## 3. Discussion

Temperate grassland heterotrophic respiration exhibits pronounced seasonality. The main findings are as follows: (1) Since 2014, the interannual patterns of GS and non-GS Rh have diverged, with non-GS Rh steadily increasing while GS Rh declined. (2) GS and non-GS Rh responses to environmental drivers were the mirror-opposite: GS Rh decreased with increasing Ta, Ts, and VPD, but increased with P and SWC; non-GS Rh showed the opposite response. Our results demonstrate that non-GS Rh has become a critical contributor to annual Rh in temperate grasslands, gradually matching and even surpassing GS Rh from 2014 onward.

### 3.1. Seasonality and Magnitude of Soil Rh

In this study, 19 temperate grassland eddy-covariance sites (2002–2021) covered a wide spatial range. The daily Rh emission rate (2.38 ± 1.47 g C m^−2^ d^−1^) fell within the reported range for temperate grasslands (0.30–5.18 g C m^−2^ d^−1^) [27,28,29]. The mean Rh during GS and non-GS was 527 ± 357 g C m^−2^ yr^−1^ and 341 ± 180 g C m^−2^ yr^−1^, respectively, accounting for 57.8 ± 14.6% and 42.2 ± 14.6% of the annual Rh, respectively. In comparison with a previous study, our findings about the non-GS Rh contribution are higher than those reported for temperate deciduous forest in Northeast China [16]. This difference is partly due to the higher temperatures in our study region, and partly attributed to methodological differences between the top-down and bottom-up measurement approaches [23].

### 3.2. Growing-Season Rh and Its Drivers

As mentioned above, in this study, GS Rh and non-GS Rh in temperate grasslands showed opposite trends after 2014, with GS Rh weakening rather than increasing (Figure 2). Typically, increasing temperature during GS enhances microbial energy availability and promotes Rh [16,30], as Rh represents the process in which organisms utilize organic substrates (e.g., glucose and fats) as energy sources, with microorganisms serving as the primary decomposers of SOC in terrestrial ecosystems [31,32,33]. Microbial communities possess both active and dormant physiological states, and only active microorganisms can decompose SOC and produce enzymes [34,35]. To maintain microbial activity, suitable soil temperature, adequate moisture, and sufficient substrate are required [36,37,38]. In this study, however, GS Rh declined because its main drivers shifted to precipitation and SWC (Figure 4 and Figure 5). Moisture, a key driver of Rh, directly or indirectly influences microbial and enzyme activity, thereby regulating SOC decomposition [39]. Since 2014, precipitation at these sites has decreased (Figure 1), while elevated temperatures have enhanced soil water evaporation, further reducing SWC (Figure 1) and making microbial activity more sensitive to moisture limitation. Ultimately, high temperatures induced soil drought, which suppressed the ability of microorganisms to decompose SOC and plant litter [40]. Moreover, grassland degradation [1] has reduced plant carbon sequestration capacity (Figure S3), thereby lowering the availability of carbon substrates for microbial metabolism, which further declined GS Rh.

### 3.3. Non-Growing Season Rh and Its Drivers

The increase in the contribution of non-GS Rh to annual Rh is due to the combined effect of decreasing SWC (Figure 1), substrate quality, and adequate carbon source. Specifically, SWC, as the most important driver of Rh in the non-GS (Figure 4 and Figure 5), was significantly negatively correlated with SWC (R = −0.76, *p* < 0.001). This phenomenon suggests that high soil moisture content reduces Rh levels by limiting oxygen diffusion and inhibiting the decomposition activities of aerobic microorganisms [39,41]. However, non-GS SWC has shown a fluctuating downward trend in recent years (Figure 1), indicating that soil moisture is in a more suitable range for microbial activity, thus promoting the enhancement of Rh. Additionally, grassland degradation may increase the soil C:N ratio [42], reduce microbial carbon-use efficiency [43], and lead to more carbon being released into the atmosphere in the form of respiration. Under the conditions of rising temperature and suitable moisture, there are still significant carbon sources for microbial decomposition during non-GS (Table S1), which enhances Rh output.

## 4. Materials and Methods

### 4.1. Study Area

**Figure 6:**
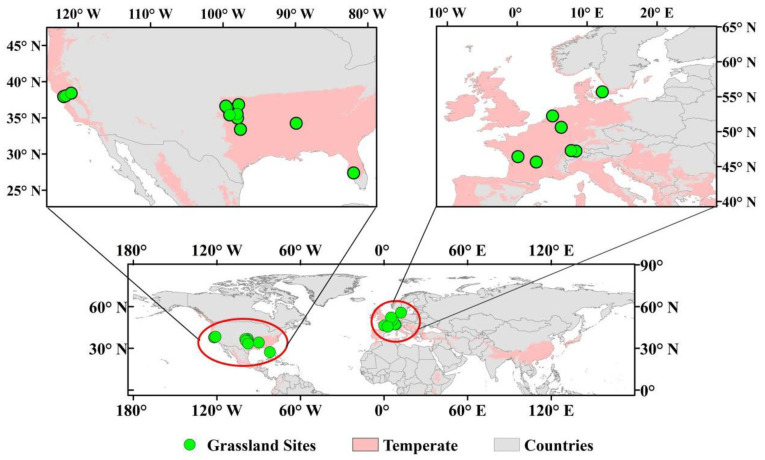
Spatial distribution of 19 northern temperate grasslands.

In this study, long-term eddy flux observations were obtained from public flux tower datasets such as FLUXNET2015, AmeriFlux, ChinaFlux, and ICOS. Based on the Köppen climate classification [44], 19 flux observation sites in Europe and the United States were selected by screening the northern temperate grassland ecosystems (Figure 6), with data from FLUXNET2015 Tier 1 [45] and AmeriFlux FLUXNET (https://ameriflux.lbl.gov/ (accessed on 1 May 2025)) (Table S1). Therefore, the temperate grasslands in this study only represent European and American prairies.

The geographical distribution of the selected temperate grassland sites ranged from 5.07° W to 121.98° E and 27.38° N to 55.69° N. The study region exhibits an annual average temperature of 8–23 °C, with precipitation ranging from 41 to 591 mm and a saturated water vapor pressure deficit (VPD) of 3–11 hPa. The soil organic carbon (SOC) content in the 0–30 cm layer, derived from SoilGrids [46], averages 26.88 ± 21.89 g kg^−1^ (Table S1). Most of the stations have a period of 2–5 years, and only two stations have a continuous observation record of more than 10 years. The number of sites has increased significantly since 2002 (Figure S1).

### 4.2. Calculation of Seasonal Rh

To explore the interannual variation pattern of Rh contribution of seasonal Rh in temperate grasslands, a top-down approach was used to calculate heterotrophic (or microbial) respiration ($R_{h}$, g C m^−2^ d^−1^) based on daily scale data from FLUXNET2015 and AmeriFlux eddy flux stations, combined with MODIS GPP and NPP data products [23,47]. The calculation formula is as follows:

(1) $$R_{h}=\mathrm{RECO}-R_{a}=\mathrm{RECO}-(1-{\mathrm{CUE}}_{p})\times \mathrm{GPP}$$

(2) $${\mathrm{CUE}}_{p}={\mathrm{CUE}}_{0}+\frac{0.1\times (\mathrm{GPP}-\bar{\mathrm{GPP}})}{\mathrm{SD}(\mathrm{GPP}-\bar{\mathrm{GPP}})}$$

where *RECO* is the ecosystem respiration (g C m^−2^ d^−1^), $R_{a}$ is the plant autotrophic respiration (g C m^−2^ d^−1^), *GPP* is the gross primary productivity (g C m^−2^ d^−1^), and ${CUE}_{p}$ is the plant carbon use efficiency (Unitless). *RECO* and *GPP* are obtained from the net ecosystem exchange observed at the site by the nighttime flux splitting method [48]. ${CUE}_{p}$ is calculated as the ratio of net primary productivity (NPP) to *GPP*, which were obtained from the MOD17A2 and MOD17A3 products (spatial resolution: 500 m) [49,50]. ${CUE}_{0}$ was calculated as the annual ratio of NPP to *GPP*, $\bar{GPP}$ is the average annual *GPP*, and the coefficient of variation of monthly GPP $\left(GPP-\bar{GPP}\right)$ was used to indicate its seasonal fluctuation. Negative values encountered during the calculation process were excluded from analysis.

Due to the temporal coverage of MOD17A2 GPP and MOD17A3 NPP data (from 18 February 2000 to 31 December 2021), and considering that most of the site-level observations began in 2002, the analysis period of this study was set to 2002–2021, totaling 20 years. The growing season was defined as May to September, and the non-growing season as January to April and October to December [13,51,52]. The contributions of GS and non-GS Rh to annual cumulative Rh were calculated, along with their interannual variation patterns. Given the differences in the observation periods among the sites, Z-score standardization was applied to Rh contributions within each site to eliminate the effects of absolute value differences and to better reflect interannual variability at the regional scale.

### 4.3. Data Processing of Hydrothermal Variables

To investigate the response of seasonal Rh contributions to hydrothermal conditions, five key hydrothermal variables were selected: air temperature (Ta), precipitation (P), vapor pressure deficit (VPD), shallow soil temperature (Ts), and shallow soil water content (SWC). All hydrothermal data were obtained from in situ site measurements. For missing Ts and SWC values during the period of 2002–2021, this study extracted 0–7 cm depth data from the ERA5-Land reanalysis dataset daily [53], based on site geographic coordinates. Missing values were gap-filled by establishing linear regression models between the measured and reanalysis data, yielding coefficients of determination (R^2^) of 0.91 for soil temperature and 0.53 for soil moisture (Figure S2).

Finally, Spearman correlation analysis was conducted to assess the relationships between the five selected variables and the seasonal Rh contributions. To account for the effects of other variables, partial correlation analysis was also applied. In addition, we employed the eXtreme Gradient Boosting (XGBoost) model [54] to further explore the non-linear relationships and assess the relative importance of each variable in predicting Rh contributions. The dataset was randomly divided into a training set (70%) and a validation set (30%). The model was trained to minimize RMSE between the observed and predicted seasonal Rh contributions. To avoid overfitting, we applied column sampling by setting both the subsample and colsample_bytree parameters to 0.8. An early stopping strategy was also implemented, with the number of boosting rounds capped at 10 if validation performance did not improve. To evaluate model performance, both R^2^ and RMSE were used as metrics. All data processing and analysis were performed using R Statistical Software (v4.5.1; R Core Team 2025) [55].

## 5. Conclusions

Temperate grasslands are among the most sensitive and vulnerable ecosystems to global warming, storing substantial amounts of soil organic carbon. Under the background of global change, soil respiration in temperate grasslands has been increasing, accelerating SOC decomposition rates [12,56,57]. In this study, soil heterotrophic respiration was divided into growing and non-growing seasons to better examine its feedback with climate change. Although the site-level data used in this study did not all span the full period from 2002 to 2021, only two eddy covariance sites had observation records longer than 10 years. To include a broader range of climatic conditions, we applied mean value standardization to eliminate site-specific differences. The results revealed that the annual increase in Rh in temperate grasslands mainly originated from the non-growing season, where rising temperatures and reduced soil moisture enhanced microbial activity. In contrast, warming and drying trends during the growing season suppressed microbial respiration. These seasonal asymmetries may ultimately shift the carbon sink–source dynamics of temperate grasslands, altering their role in the global carbon cycle.

Compared to previous studies on soil respiration responses to warming and drying, our study provides a new perspective by identifying a convergence pattern of Rh contribution during both GS and non-GS under climate change, utilizing a long-term, multi-site dataset derived from MODIS and eddy covariance measurements. Admittedly, due to limitations in the datasets and Rh measurement techniques, site-level observations of Rh were not available at flux tower sites in this study. As a result, the Rh estimates derived from the top-down approach may involve uncertainties. In the future, if long-term Rh measurements can be obtained at flux tower sites, it would be valuable to validate and refine these estimates against observations. Furthermore, although site-level influences were minimized through data standardization, most flux sites have relatively short observation periods. Thus, calculating average trends over the past two decades across all sites may introduce some uncertainty. Future studies would benefit from more long-term data to better capture interannual variation patterns. Additionally, due to the lack of in situ SWC measurements at the study sites, this study employed a linear regression model based on ERA5-Land data for gap-filling. However, the relatively low coefficient of determination (R^2^ = 0.53) inevitably introduces uncertainty into the imputed values. Finally, since microbial decomposition of SOC is the main source of Rh [31,32,33], while acknowledging that this assumption does not account for potential contributions from soil fauna-mediated SOC decomposition processes.

## Data Availability

The following supporting data are publicly available: flux tower site data can be accessed at https://fluxnet.org/ (accessed on 1 May 2025), MODIS GPP and NPP products are available for download at https://www.earthdata.nasa.gov/ (accessed on 1 May 2025), and soil temperature and moisture data are provided at https://cds.climate.copernicus.eu/ (accessed on 17 May 2025).

## Supplementary Materials

The following supporting information can be downloaded at: https://www.mdpi.com/article/10.3390/plants14162590/s1. Figure S1: Annual distribution of observation data of 19 temperate grassland vortex stations. Figure S2: The linear relationship between the EAR5-Land dataset and the measured soil temperature (left) and water content (right) at the vortex site. Figure S3: The z-score normalized gross primary productivity (GPP) across global flux tower sites. Table S1: Basic information on 19 northern temperate grasslands integrated.
