# Supplementary material for: Converging Patterns of Heterotrophic Respiration Between Growing and Non-Growing Seasons in Northern Temperate Grasslands
Source: Plants (Basel). 2025 Aug 20;14(16):2590. doi: 10.3390/plants14162590 (PMC12389485; doi:10.3390/plants14162590)
Supplement: Supplementary file 1 [file plants-14-02590-s001.zip › plants-3804319-supplementary.pdf]

Supplement Data

Table S1. Basic information on 19 northern temperate grasslands integrated. MAT: multi-year average temperature; MAP: multi-year average precipitation; VPD: Saturated Water Air Pressure Difference; SOC: soil organic carbon.

ID	Site Name	Longitude (°)	Latitude (°)	Start Year	Stop Year	Data Source	MAT (℃)	MAP (mm)	VPD (hPa)	SOC (g kg^{-1})
1	CH-Cha	8.410	47.210	2005	2014	FLUXNET2015	9	568	3	41.65
2	CH-Oe1	7.732	47.286	2002	2008	FLUXNET2015	9	428	3	37.60
3	DE-RuR	6.304	50.622	2011	2014	FLUXNET2015	8	174	3	58.28
4	DK-Eng	12.192	55.691	2005	2008	FLUXNET2015	9	166	3	26.10
5	NL-Hor	5.071	52.240	2004	2011	FLUXNET2015	11	414	3	90.82
6	US-ARc	-98.040	35.546	2005	2006	FLUXNET2015	16	76	8	13.58
7	US-Goo	-89.874	34.255	2002	2006	FLUXNET2015	16	357	5	11.43
8	US-A32	-97.820	36.819	2015	2017	AmeriFlux	17	122	9	11.93
9	US-AR1	-99.420	36.427	2009	2012	AmeriFlux	15	95	9	9.88
10	US-AR2	-99.598	36.636	2009	2012	AmeriFlux	15	89	10	3.35
11	US-ARb	-98.040	35.550	2005	2006	AmeriFlux	17	77	9	13.32
12	US-ARc	-98.040	35.547	2005	2006	AmeriFlux	17	83	9	13.58
13	US-CGG	-121.976	37.938	2019	2021	AmeriFlux	16	41	10	14.57
14	US-ONA	-81.951	27.384	2016	2022	AmeriFlux	23	293	7	30.18
15	US-Sne	-121.755	38.037	2016	2020	AmeriFlux	15	79	7	47.23
16	US-Snf	-121.733	38.040	2018	2020	AmeriFlux	15	45	9	43.95
17	US-Var	-120.951	38.413	2000	2021	AmeriFlux	16	591	11	13.38
18	US-xAE	-99.059	35.411	2017	2021	AmeriFlux	16	343	9	11.88
19	US-xCL	-97.570	33.401	2017	2021	AmeriFlux	18	147	9	17.97

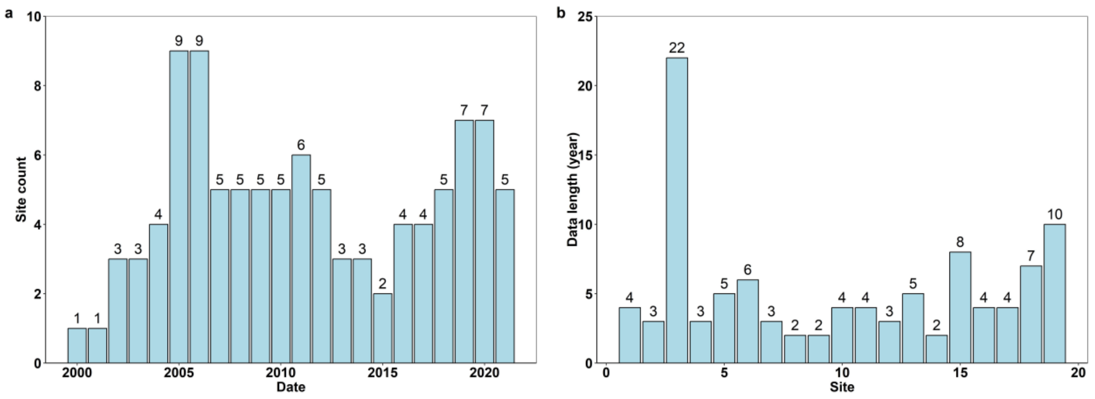

Figure S1. Annual distribution of observation data of 19 temperate grassland vortex stations.

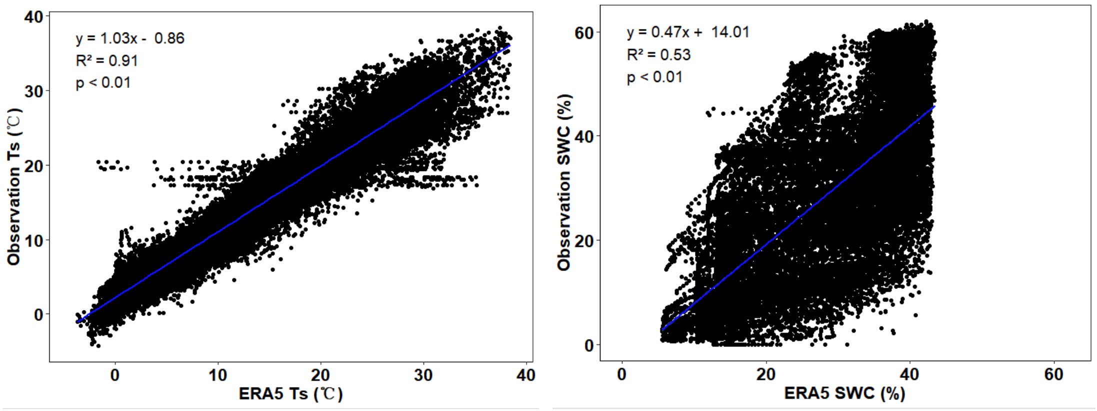

Figure S2. The linear relationship between the EAR5-Land dataset and the measured soil temperature (left) and water content (right) at the vortex site. Ts is shallow soil temperature; SWC is the shallow soil moisture content.

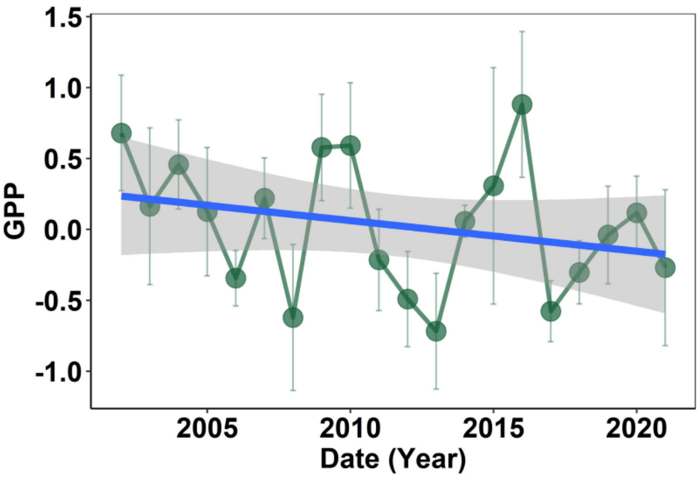

Figure S3. The z-score normalized gross primary productivity (GPP) across global flux tower sites. The error bar is standard error. Solid lines represent trend fits, and shaded area indicates the 95% confidence intervals.
